# Epidemiological profile and determinants of whole blood heavy metal levels in occupationally exposed populations: a cross-sectional study in Hunan Province, China

**DOI:** 10.3389/fpubh.2025.1635236

**Published:** 2025-07-25

**Authors:** Lina Wen, Lezhou Zhou, Xiaochao Zhu, Yong Mei

**Affiliations:** ^1^Public Health and Preventive Medicine, Wuhan University of Science and Technology, Wuhan, China; ^2^Central Laboratory, Hunan Prevention and Treatment Institute for Occupational Diseases, Changsha, China

**Keywords:** heavy metal exposure, occupationally exposed populations, exposure determinants, Hunan Province, influencing factors

## Abstract

**Objective:**

This study aims to characterize current whole blood levels of heavy metals including lead (Pb), cadmium (Cd), mercury (Hg), and arsenic (As) among occupational populations in Hunan Province, China, and identify exposure determinants to inform health management strategies.

**Methods:**

A cross-sectional study was conducted on 2,991 occupational workers. Demographic data, occupational exposure history, and lifestyle habits were collected. Whole blood samples were analyzed via atomic absorption spectrophotometry for Pb and Cd levels, and atomic fluorescence spectrophotometry for Hg and As concentrations. Multiple linear regression was used to identify exposure predictors, and K-means clustering to categorize exposure patterns.

**Results:**

Elevated exceedance rates were observed for all metals, with Hg showing the highest rate (17.39%). Significantly higher blood metal levels (*p* < 0.05) were associated with males, age > 50 years, employment duration > 20 years, mining occupations, residence in Chang-Zhu-Tan, smoking, and drinking. Regression analyses revealed gender, age, employment duration, occupation type, and smoking as significant predictors of Pb and Cd levels (*p* < 0.05); gender, age, employment duration, and drinking for Hg (*p* < 0.05); and gender, age, employment duration, occupation type, and geographic region for As (*p* < 0.05). K-means clustering stratified participants into low-, medium-, and high-exposure groups, with the latter exhibiting markedly elevated metal levels (*p* < 0.05), including some samples exceeding occupational exposure limits.

**Conclusion:**

Whole blood heavy metal levels in Hunan occupational populations are significantly influenced by gender, age, employment duration, occupation type, and geographic factors. Targeted exposure mitigation and enhanced biomonitoring are urgently needed for high-risk subgroups.

## 1 Introduction

Heavy metal pollution has emerged as a global environmental and public health challenge driven by rapid industrialization and urbanization. Characterized by persistence, bioaccumulation, and high toxicity, heavy metals such as lead (Pb), cadmium (Cd), mercury (Hg), and arsenic (As) infiltrate human systems through atmospheric, aquatic, and terrestrial pathways, posing severe health threats (1, 2). Cumulative evidence indicates that occupational populations chronically exposed to these metals may face elevated risks of neurological impairment, renal dysfunction, cardiovascular disorders, and even carcinogenesis (3). For instance, an increase in blood Pb levels is closely related to cognitive decline and anemia (4). Exposure to Cd can cause renal damage and bone diseases (5). Higher blood Hg levels are associated with neurotoxicity and an elevated risk of cardiovascular diseases (6). Blood As exposure is linked to various diseases like skin lesions and cancers (7). Notably, occupational cohorts always exhibit higher blood metal burdens due to prolonged workplace exposure, correlating with increased morbidity and mortality rates in epidemiological studies (8).

Despite advancements in assessing occupational heavy metal exposure and its influencing factors, existing research remains fragmented by its focus on single industries or localized regions. Comprehensive evaluations integrating multi-metal co-exposure analyses and influencing factors are limited. This gap is particularly relevant in Hunan Province, a pivotal industrial hub in China with a substantial workforce engaged in mining, manufacturing, and construction. Inadequate environmental safeguards and insufficient occupational protection awareness have exacerbated heavy metal exposure risks among these populations (9, 10). Although efforts for occupational disease prevention have intensified in recent years, systematic investigations into whole blood heavy metal levels across diverse occupational groups remain sparse, particularly regarding multi-metal assessments and mechanistic exploration of influencing factors.

This study aims to investigate the distribution patterns of whole blood heavy metals (Pb, Cd, Hg, As) among occupationally exposed populations in Hunan Province and elucidate determinants spanning demographic traits, occupational exposure characteristics, and lifestyle behaviors. By integrating multi-metal biomonitoring with multivariate analysis, our findings will clarify regional exposure profiles and inform evidence-based occupational health strategies. These efforts align with national objectives to mitigate workplace hazards and advance the implementation of occupational disease prevention and control frameworks.

**Figure 1:**
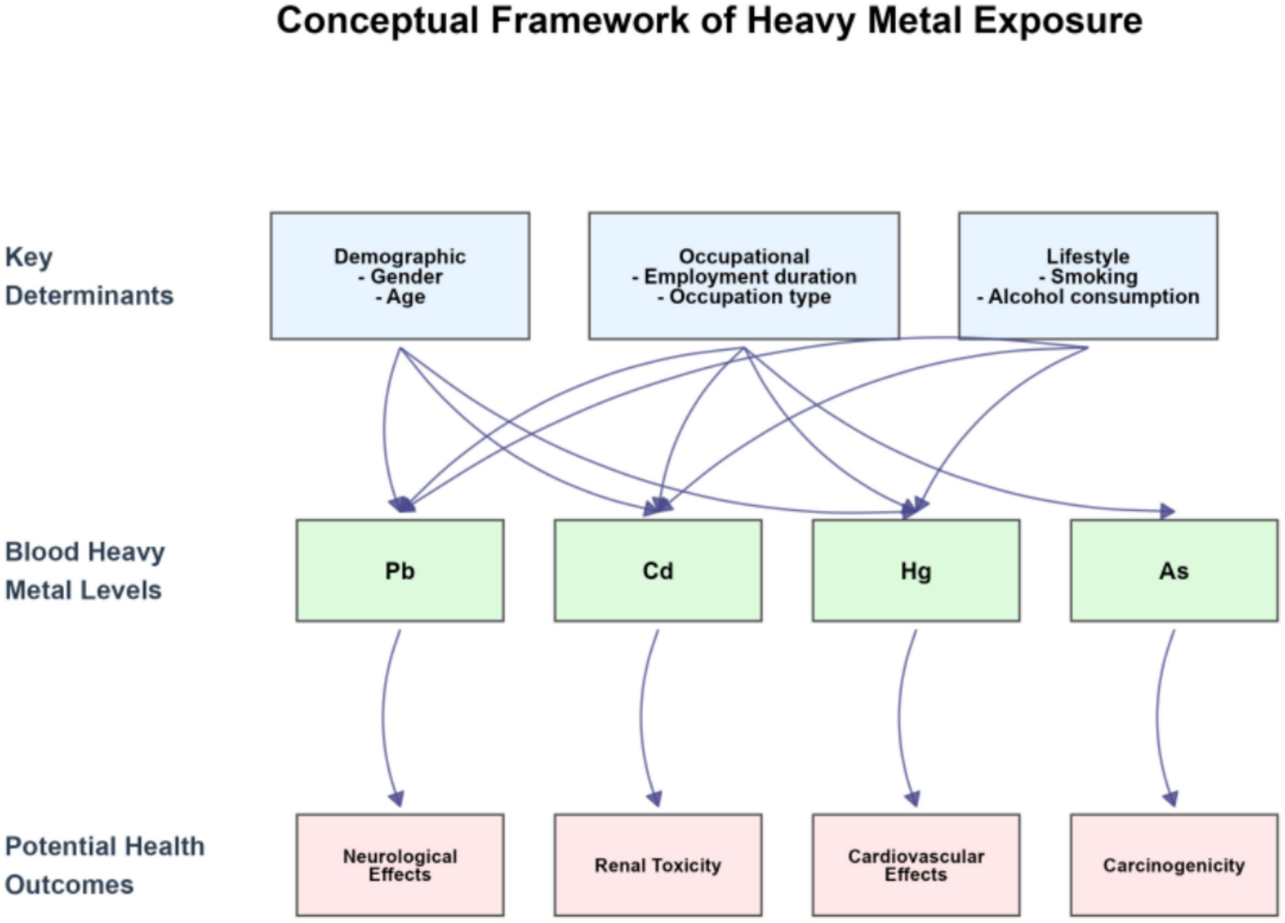
Conceptual framework of heavy metal exposure in occupational populations.

To intuitively present the core logic of “heavy metal exposure among occupational populations” that this study focuses on, we constructed a conceptual framework diagram of heavy metal exposure (Figure 1), which clearly sorts out the association chain of “key determining factors → whole - blood heavy metal levels → potential health outcomes.” Among them, demographic characteristics such as gender and age, occupational attributes such as employment duration and occupation type, and lifestyle habits such as smoking and alcohol consumption constitute the key determining factors of exposure; these factors affect the levels of heavy metals such as Pb, Cd, Hg, and As in whole blood through pathways like occupational contact and environmental exposure; and the long - term accumulation of heavy metals may induce health hazards such as neurotoxicity, nephrotoxicity, cardiovascular damage, and carcinogenicity.

## 2 Materials and method

### 2.1 Study population

This study employed stratified random sampling to recruit participants. Based on the industry demographics published by the Hunan Provincial Bureau of Statistics, the occupational populations were stratified by occupation type (mining, construction, manufacturing, agriculture, and others) and geographic region (Chang-Zhu-Tan, Xiangnan, Xiangxi, Dongting Lake). Within each stratum, participating enterprises were randomly selected from official registries, and employees were further randomly sampled within enterprises according to employment duration and gender distributions. Ultimately, 2,991 participants were enrolled for a cross-sectional survey, with sample characteristics closely reflecting the occupational structure of Hunan Province to ensure representativeness. Eligible participants met the following inclusion criteria: (1) age ≥ 18 years with current employment in recognized occupational sectors across Hunan Province; and (2) minimum employment duration of 1 year in their current position. Exclusion criteria comprised: (1) pre-existing chronic conditions affecting heavy metal metabolism (hematological disorders, renal dysfunction, or hepatic diseases); (2) history of heavy metal chelation therapy or use of medications known to influence heavy metal metabolism within 3 months prior to enrollment; and (3) pregnancy, lactation, or childhood. The study protocol received ethical approval from the Hunan Prevention and Treatment Institute for Occupational Diseases (Approval No. 2025051301).

### 2.2 Study methods

Demographic characteristics (gender, age, employment duration), occupational exposure profiles (occupation type, geographic region), and lifestyle factors (smoking status, drinking status) were retrospectively extracted from historical physical examination records. Concurrently, whole blood samples were collected from participants for quantification of blood Pb, Cd, Hg, and As concentrations.

### 2.3 Data and specimen collection

Certified healthcare professionals collected 5 mL of antecubital venous blood from each participant. Blood samples were immediately transferred to anticoagulant-containing vacuum tubes, gently inverted to ensure proper mixing, and stored at 2–8°C. All specimens were processed within 24 h to minimize pre-analytical variability. Quantification of whole blood heavy metal concentrations was performed using atomic absorption spectrophotometry (11) for Pb and Cd, and atomic fluorescence spectrophotometry (12) for Hg and As.

### 2.4 K-means cluster analysis

K-means cluster analysis, an unsupervised machine learning algorithm, was employed to classify occupational populations based on whole blood heavy metal exposure profiles. Blood Pb, Cd, Hg, and As concentrations were selected as clustering variables, with raw data normalized. The optimal cluster number was determined using the Elbow method, which identified a definitive inflection point at *k* = 3. Participants were subsequently stratified into three distinct exposure categories: Low-exposure cluster: Characterized by blood concentrations below occupational exposure limits (OELs) for all metals (Pb ≤ 200 μg/L, Cd ≤ 2 μg/L, Hg ≤ 5 μg/L, and As ≤ 10 μg/L). Medium-exposure cluster: Defined by ≥ 1 parameter meeting or marginally exceeding OELs (Pb 200–400 μg/L, Cd 2–5 μg/L, Hg 5–15 μg/L, and As 10–30 μg/L). High-exposure cluster: Comprising participants with ≥ 1 parameter substantially exceeding OELs (Pb > 400 μg/L, Cd > 5 μg/L, Hg > 15 μg/L, and/or As > 30 μg/L).

### 2.5 Statistical analysis

Data processing and statistical analyses were conducted using SPSS Statistics 26.0 and R programming language. After normality assessment, normally distributed continuous variables were expressed as mean ± standard deviation (x̄ ± s) and analyzed using independent samples t-tests (two-group comparisons) or one-way analysis of variance (ANOVA), with LSD-t test for pairwise comparisons or Tukey’s method for correction. Non-normally distributed variables were reported as median with interquartile range [M (P25, P75)] and evaluated via Mann–Whitney U tests or Kruskal-Wallis rank sum tests. Categorical variables were presented as *n* (%) and compared using χ^2^ tests or Fisher’s exact probability tests as appropriate. Multivariate linear regression models were constructed to assess the independent effects of key predictors, including gender, age, employment duration, occupation type, geographic region, smoking status, and drinking status, on whole blood heavy metal levels. All covariates were simultaneously included in the fully adjusted models. K-means clustering was used to quantify exposure pattern heterogeneity. A *p*-value < 0.05 was considered statistically significant.

## 3 Results

### 3.1 Current status of whole blood heavy metals in occupational populations

A cohort of 2,991 occupationally exposed individuals was analyzed for whole blood concentrations of Pb, Cd, Hg, and As. The results revealed significant exceedance of OELs for all four heavy metals, with blood Hg demonstrating the highest exceedance rate (Table 1). These findings highlight substantial exposure risks that need targeted mitigation strategies in high-risk occupational environments.

**Table 1:** Blood concentrations and exceedance rates of Pb, Cd, Hg, and As in the study cohort.

Heavy metal type	Concentration (μg/L)	Number of exceedances (*n*)	Exceedance rate (%)
Blood Pb	229.3 (158.7–317.8)	355	11.87
Blood Cd	2.1 (1.1–3.6)	352	11.77
Blood Hg	7.5 (4.0–12.8)	520	17.39
Blood As	13.3 (6.9–23.9)	462	15.45

### 3.2 Comparison of whole blood heavy metal levels across demographic subgroups

Demographic stratification of the occupational cohort (*n* = 2,991) demonstrated the following distributions: gender (male: *n* = 1,521; female: *n* = 1,470), age (< 30 years: *n* = 763; 30–40 years: *n* = 768; 41–50 years: *n* = 705; > 50 years: *n* = 755), employment duration (< 5 years: *n* = 762; 5–10 years: *n* = 770; 11–20 years: *n* = 717; > 20 years: *n* = 742), occupation type (mining: *n* = 626; construction: *n* = 597; manufacturing: *n* = 537; agriculture: *n* = 625; others: *n* = 606), geographic region (Chang-Zhu-Tan: *n* = 799; Xiangnan: *n* = 745; Xiangxi: *n* = 727; Dongting Lake: *n* = 720), smoking status (smoking: *n* = 1,468; non-smoking: *n* = 1,523), and drinking status (drinking: *n* = 1,485; non-drinking: *n* = 1,506).

**Figure 2:**
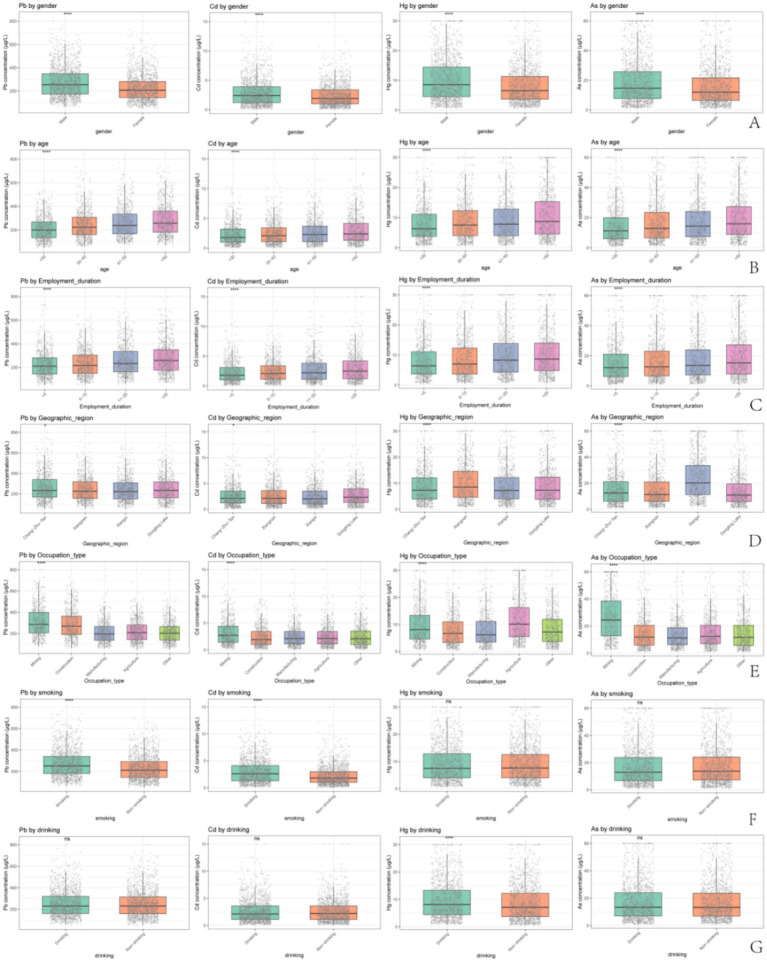
Comparative whole blood heavy metal concentrations across demographic subgroups. **(A)** Comparison of blood lead, cadmium, mercury, and arsenic levels between male and female participants. **(B)** Comparison of heavy metal levels across age groups (< 30, 30–40, 41–50, > 50 years). **(C)** Comparison of heavy metal levels across employment duration groups (< 5, 5–10, 11–20, > 20 years). **(D)** Comparison of heavy metal levels across occupational categories (mining, construction, manufacturing, agriculture, others). **(E)** Comparison of heavy metal levels across geographic regions (Chang-Zhu-Tan, Xiangnan, Xiangxi, Dongting Lake). **(F)** Comparison of heavy metal levels between smokers and non-smokers. **(G)** Comparison of heavy metal levels between drinkers and non-drinkers. ns = non-significant (*p* > 0.05), * = *p* < 0.05; **** = *p* < 0.0001.

In the gender subgroups, males had significantly higher levels of blood Pb, Cd, Hg, and As compared with females (*p* < 0.05; Figure 2A). Age-based subgroup analysis indicated that the levels of heavy metals increased with age, with the > 50-year group showing the highest levels (*p* < 0.05; Figure 2B). As for employment duration, longer employment was associated with higher levels of blood Pb, Cd, Hg, and As, with maximal levels in the > 20-year group (*p* < 0.05; Figure 2C). Among occupational categories, mining workers exhibited the highest Pb, Cd, Hg, and As levels, while agricultural workers showed peak blood Hg concentrations (*p* < 0.05; Figure 2D). Geographically, the highest blood Pb levels were observed in Chang-Zhu-Tan, Cd in Dongting Lake, Hg in Xiangnan, and As in Xiangxi (*p* < 0.05; Figure 2E). In terms of lifestyle behaviors, smokers had increased blood Pb and Cd compared with non-smokers, while drinkers demonstrated higher blood Hg levels than non-drinkers (*p* < 0.05; Figures 2F,G).

**Figure 3:**
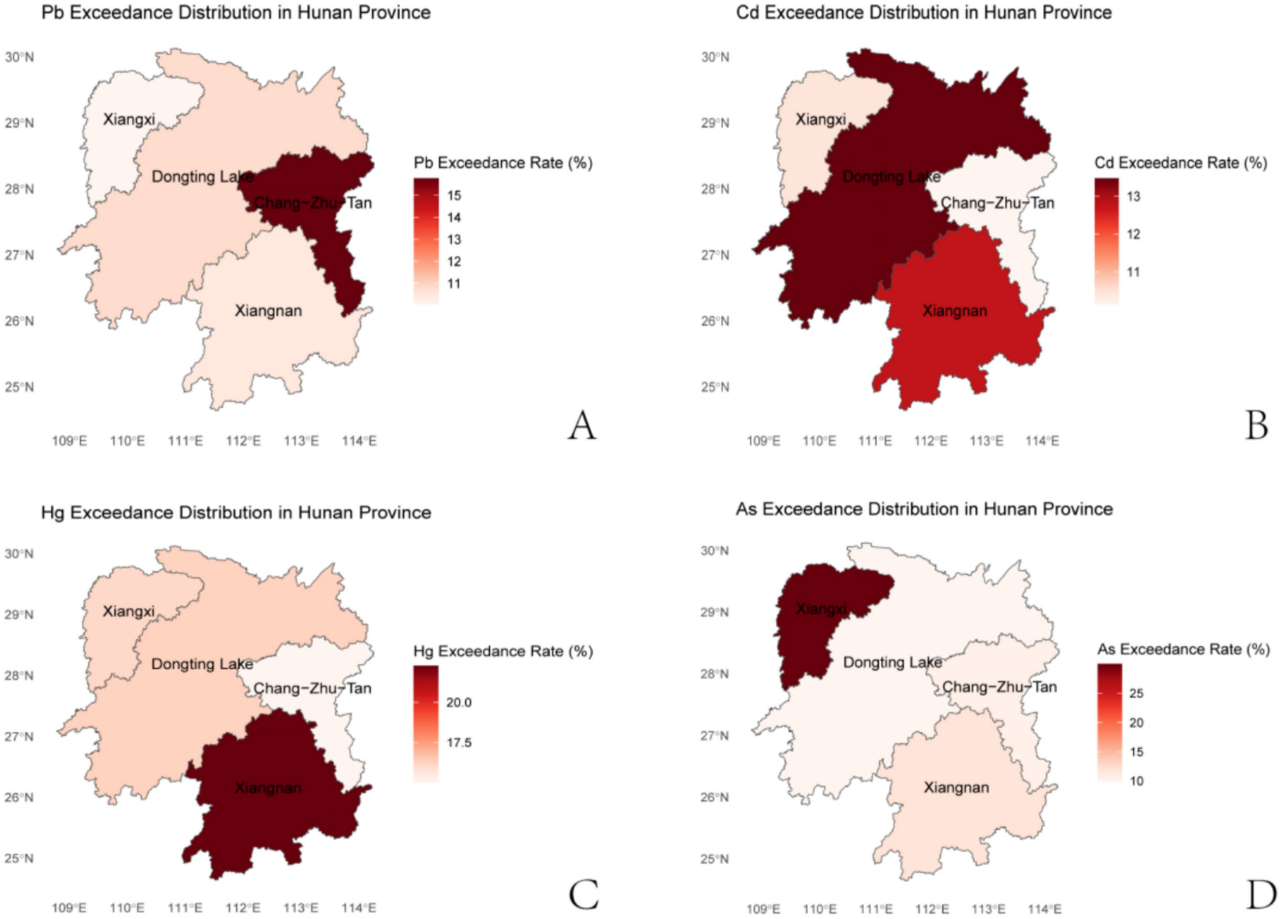
Geographic distribution map of heavy metal exposure (Hunan Province). Panel **(A–D)** represent the spatial differences in the average exceedance rates of key heavy metals (Pb, Hg, Cd, As) in whole blood across Chang-Zhu-Tan, Xiangnan, Xiangxi, and Dongting Lake regions, respectively. The color gradient reflects the magnitude of exceedance rates (low to high), with data derived from the statistical analysis of 2,991 occupational participants. Regional boundaries are based on administrative divisions of Hunan Province, and the distribution patterns correspond to localized industrial activities and environmental pollution sources.

The geographic distribution map confirms that heavy metal exposure is not uniformly distributed but rather tied to regional industrial and environmental characteristics, reinforcing the role of geographic factors as key determinants of occupational exposure (Figures 3A–D).

### 3.3 Multivariate linear regression analysis of determinants influencing whole blood heavy metal levels

Separate multivariate linear regression models evaluated demographic, occupational, and behavioral covariates (categorized in Table 2) as independent predictors of whole blood Pb, Cd, Hg, and As concentrations. The regression models identified gender, age, employment duration, occupation type, and smoking status as significant determinants of blood Pb levels (*p* < 0.05; Table 3). Similarly, blood Cd concentrations were independently associated with gender, age, employment duration, occupation type, and smoking status (*p* < 0.05; Table 4). For blood Hg, significant predictors included gender, age, employment duration, and drinking status (*p* < 0.05; Table 5), whereas blood As levels were significantly influenced by gender, age, employment duration, occupation type, and geographic region (*p* < 0.05; Table 6).

**Table 2:** Variable categorization and coding schema.

Variable	Category and assignment
Gender	1 = Male, 2 = Female
Age	1 = < 30, 2 = 30–40, 3 = 41–50, 4 = > 50
Employment duration	1 = < 5, 2 = 5–10, 3 = 11–20, 4 = > 20
Occupation type	1 = Mining, 2 = Construction, 3 = Manufacturing, 4 = Agriculture, 5 = Others
Geographic region	1 = Chang-Zhu-Tan, 2 = Xiangnan, 3 = Xiangxi, 4 = Dongting Lake
Smoking status	1 = Smoking, 2 = Non-smoking
Drinking status	1 = Drinking, 2 = Non-drinking

**Table 3:** Multivariate linear regression analysis of factors influencing blood Pb levels.

Variable	*B*	*β*	*t*	*p*	95%CI for *B*		*R^2^*	*F*
					Lower limit	Upper limit		
Gender	−49.234	−0.200	−12.530	< 0.001	−56.939	−41.530	0.490	157.541
Age	23.139	0.211	13.242	< 0.001	19.713	26.565		
Employment duration	19.021	0.173	10.844	< 0.001	15.582	22.461		
Occupation type	−26.61	−0.310	−19.428	< 0.001	−29.296	−23.925		
Geographic region	1.274	0.012	0.723	0.469	−2.179	4.727		
Smoking status	−42.731	−0.174	−10.872	< 0.001	−50.437	−35.024		

**Table 4:** Multivariate linear regression analysis of factors influencing blood Cd levels.

Variable	*B*	*β*	*t*	*p*	95%CI for *B*		*R^2^*	*F*
					Lower limit	Upper limit		
Gender	−0.491	−0.118	−6.816	< 0.001	−0.632	−0.35	0.315	54.682
Age	0.238	0.129	7.427	< 0.001	0.175	0.301		
Employment duration	0.261	0.141	8.114	< 0.001	0.198	0.324		
Occupation type	−0.148	−0.102	−5.889	< 0.001	−0.197	−0.099		
Geographic region	0.040	0.021	1.226	0.220	−0.024	0.103		
Smoking status	−0.796	−0.192	−11.042	< 0.001	−0.937	−0.654		

**Table 5:** Multivariate linear regression analysis of factors influencing blood Hg levels.

Variable	*B*	*β*	*t*	*p*	95%CI for *B*		*R^2^*	*F*
					Lower limit	Upper limit		
Gender	−2.059	−0.155	−8.772	< 0.001	−2.520	−1.599	0.264	37.231
Age	0.878	0.149	8.416	< 0.001	0.674	1.083		
Employment duration	0.800	0.135	7.639	< 0.001	0.595	1.006		
Occupation type	0.117	0.025	1.428	0.153	−0.044	0.277		
Geographic region	−0.038	−0.006	−0.361	0.718	−0.244	0.168		
Drinking status	−0.917	−0.069	−3.905	< 0.001	−1.377	−0.456		

**Table 6:** Multivariate linear regression analysis of factors influencing blood As levels.

Variable	*B*	*β*	*t*	*p*	95%CI for *B*		*R^2^*	*F*
					Lower limit	Upper limit		
Gender	−2.800	−0.101	−5.813	< 0.001	−3.745	−1.856	0.324	69.900
Age	1.684	0.136	7.864	< 0.001	1.264	2.104		
Employment duration	1.378	0.111	6.408	< 0.001	0.956	1.800		
Occupation type	−2.405	−0.248	−14.32	< 0.001	−2.734	−2.075		
Geographic region	0.653	0.052	3.024	0.003	0.230	1.076		

### 3.4 K-means clustering-based typology of occupational blood heavy metal exposure

**Figure 4:**
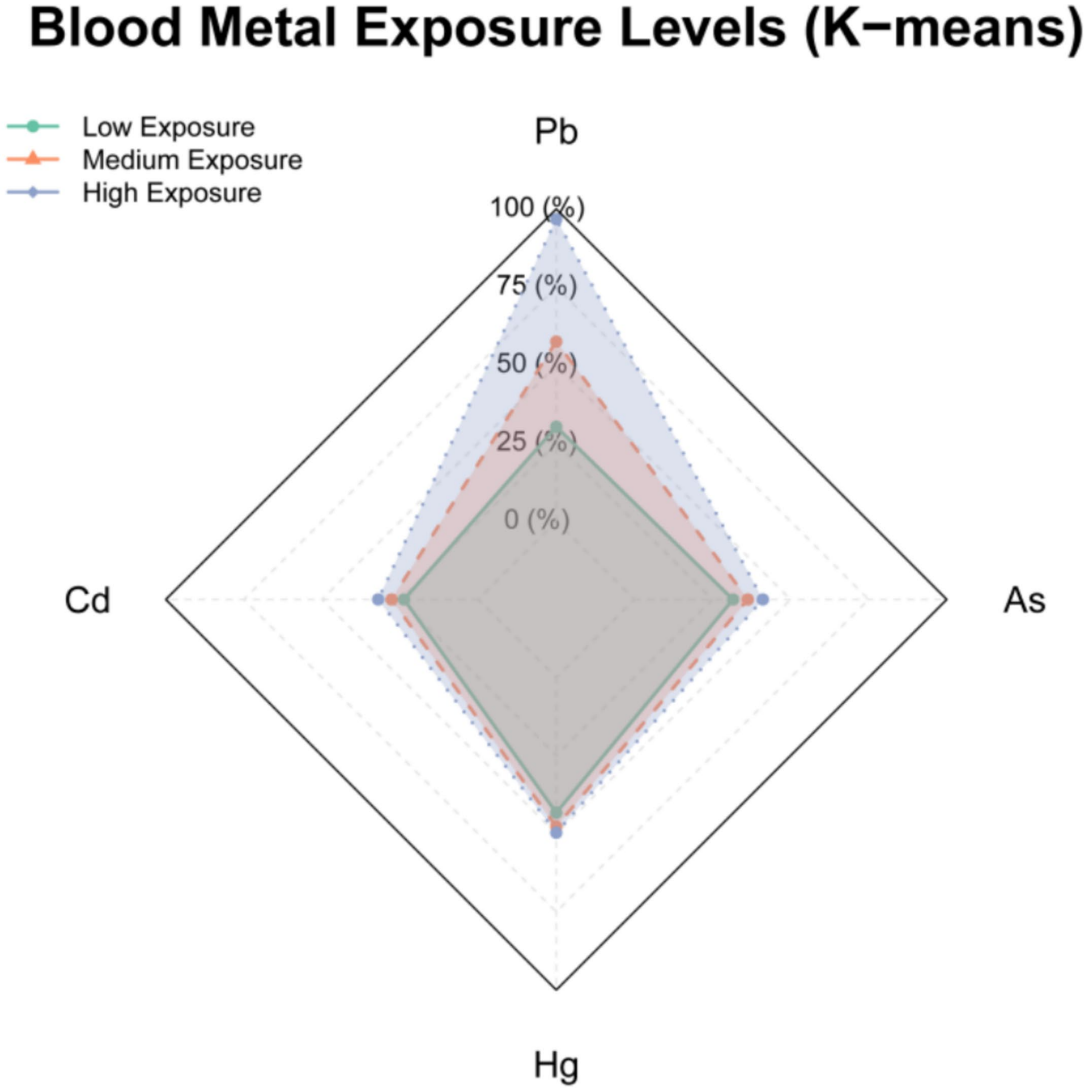
Radar plot illustrating whole blood heavy metal exposure profiles of occupational clusters based on K-means clustering.

Utilizing the K-means clustering algorithm, we stratified the occupational cohort into three distinct exposure archetypes: low-exposure (*n* = 93, 3.1%), medium-exposure (*n* = 1,614, 54.0%), and high-exposure clusters (*n* = 1,284, 42.9%). The high-exposure cluster exhibited substantially elevated blood concentrations of Pb, Cd, Hg, and As, with all participants exceeding OELs for at least one metal. In contrast, the low-exposure cluster demonstrated minimal exposure burdens of Pb, Cd, Hg, and As, with all parameters remaining far below OELs. The medium-exposure cluster displayed intermediate profiles, where some individuals approached or marginally surpassed OELs (Table 7). Radar plot visualization (Figure 4) further delineated the inter-cluster divergence in heavy metal exposure profiles.

**Table 7:** Blood heavy metal concentrations across K-means-derived exposure clusters.

Exposure cluster	Sample size (n)	Blood Pb (μg/L)	Blood Cd (μg/L)	Blood Hg (μg/L)	Blood As (μg/L)
Low-exposure	93	142.4 (108.8, 164.2)	1.0 (0.6,1.5)	3.1 (2.0, 4.2)	5.4 (3.3, 7.0)
Medium-exposure	1,614	211.85 (147.7, 272.3)	1.9 (1.0,3.0)	6.4 (3.7, 9.8)	11.4 (6.2, 17.88)
High-exposure	1,284	276.6 (192.5, 414.3)	2.7 (1.3,5.2)	11.4 (5.6, 18.33)	21.45 (9.5, 35.6)

## 4 Discussion

Heavy metals, commonly including Pb, Cd, Hg, and As, represent a class of elements with significant toxicological potential and widely present in natural environments and industrial processes. These metals enter the human body through inhalation, ingestion, and dermal contact, bioaccumulating in tissues and organs. Prolonged exposure can cause damage to the nervous system, kidneys, cardiovascular system, and immune system, and may even induce carcinogenesis (13). Occupational populations, particularly those engaged in mining, manufacturing, and construction, face disproportionate exposure risks due to inhalation of metal-laden particulates, dermal contact with contaminated surfaces, and ingestion of tainted water or food (14). Industrial processes such as smelting, metal refining, battery production, and chemical production exacerbate workplace contamination, resulting in elevated blood metal concentrations among workers compared to the general population. Studies have demonstrated that occupational exposure not only correlates with elevated heavy metal burden but also exacerbates risks of hypertension, renal dysfunction, neurological disorders, and malignancies (15, 16). In Hunan Province, a pivotal industrial hub in central-south China, diverse industries and complex occupational environments amplify heavy metal exposure risks, necessitating systematic investigations on the current status and influencing factors of heavy metal exposure among occupational populations in this region to inform targeted interventions.

This study found that whole blood heavy metal levels among the occupational populations in Hunan Province exhibited significant differences, with higher levels observed in males, individuals aged > 50 years, and those with employment duration > 20 years, particularly pronounced in the mining and manufacturing industries. These findings are generally consistent with global epidemiological trends. For example, a study on smelter workers reported that longer employment duration and male sex were associated with significantly higher blood Pb levels (17), which may be attributable to the cumulative effect of prolonged occupational exposure and sex-related differences in metal metabolism (18). Similarly, an epidemiological investigation on battery factories in low- and middle-income countries identified employment duration and sex as important determinants of blood Pb and Cd levels (19), suggesting broad applicability of these risk factors. However, some studies did not find a significant sex difference, which might be explained by differences in sample composition, a higher proportion of females in high-risk positions, or variations in detection methods (20). The results of our study facilitate the identification and targeted intervention of susceptible populations at the regional level.

In our study, mining workers exhibited significantly elevated blood Pb, Cd, and As levels, consistent with their occupational exposure characteristics. Processes including ore extraction, crushing, and transportation generate dust and wastewater containing Pb, Cd, and As, which enter the body via inhalation and dermal contact and bioaccumulate over time. This exposure pathway is well documented in prior occupational studies (21). Conversely, agricultural workers showed higher blood Hg levels, potentially reflecting residual mercury-based pesticides and contaminated irrigation water and crops in some areas (22). Additionally, elevated blood Hg among drinkers may originate from homemade alcohol production using mercury-contaminated containers in rural regions (23), indicating the role of lifestyle factors in exposure modulation.

Significant regional differences were also observed: higher blood Pb in Chang-Zhu-Tan, elevated Hg in Xiangnan, increased Cd in Dongting Lake, and higher As in Xiangxi. These patterns correspond to localized industrial activities and pollution sources. Chang-Zhu-Tan, as an industrial hub, faces heavy metal pollution mainly from industrial and vehicular emissions (24). Xiangnan’s non-ferrous metal smelting releases large amounts of Hg vapor (25). Dongting Lake shows Cd contamination from long-term fertilization and wastewater discharge (26, 27). Small-scale mining in Xiangxi contributes to As accumulation in soil and water, posing health risks to workers (28). These results confirm regional characteristics and emphasize the need for targeted, location-specific exposure interventions.

Behavioral cofactors further demonstrated synergistic interactions with smoking, drinking, and heavy metal exposure in collectively impacting human health (29, 30). In this study, smokers exhibited elevated blood Pb and Cd levels, while drinkers showed higher blood Hg concentrations. These findings suggest that smoking may contribute to Pb and Cd bioaccumulation either through both direct inhalation of metal-containing tobacco smoke and enhanced respiratory mucosal permeability (31). The elevated Hg observed among drinkers may relate to artisanal alcohol contamination or storage in Hg-tainted vessels, further exacerbated by alcohol-induced hepatic impairment that diminishes Hg clearance capacity (32). These findings emphasize the critical need to integrate lifestyle modification strategies into occupational health programs.

Multivariate linear regression analysis confirmed that gender, age, employment duration, occupation type, smoking, and drinking were significant determinants of whole blood heavy metal levels. These findings underscore that heavy metal exposure among occupational populations in Hunan Province is not only linked to workplace hazards but also influenced by individual characteristics and lifestyle habits. K-means clustering analysis further identified a high-exposure subgroup with markedly elevated blood Pb, Cd, Hg, and As concentrations, with some samples reaching or even exceeding OELs. This subgroup warrants urgent intervention through comprehensive mitigation strategies: (1) regular whole blood heavy metal screening and long-term health surveillance for high-risk occupations (e.g., mining, manufacturing) to enable early detection and intervention; (2) enhanced health education to raise awareness of heavy metal hazards and promote self-protection practices; (3) implementation of advanced pollution control technologies (e.g., waste gas scrubbers, wastewater treatment systems, dust suppression equipment) to minimize workplace contamination, coupled with systematic environmental monitoring of soil, water, and air quality; (4) provision and enforcement of certified personal protective equipment, including dust-proof masks, gloves, and protective clothing; (5) lifestyle interventions to reduce smoking and alcohol consumption; and (6) dietary interventions incorporating antioxidants (vitamins C/E, selenium) to counteract heavy metal-induced toxicity (33).

## 5 Implications and limitations

This study characterized heavy metal exposure patterns among occupational populations in Hunan Province, China. Through large-scale sample analysis, key factors influencing whole blood heavy metal levels were identified, providing important evidence to support the development of targeted occupational health protection measures. However, this study has several limitations. First, due to its cross-sectional design, causal relationships cannot be established. Second, despite the large sample size, the coverage of occupation types and geographic regions was limited, which may affect the representativeness of our findings. Third, the detection limits for some heavy metals were relatively low, potentially introducing measurement errors in low-concentration samples. Finally, potential confounding factors such as residential environmental exposure and genetic susceptibility were not adequately considered. Future studies should incorporate these factors to achieve a more comprehensive understanding of the determinants of heavy metal exposure. Furthermore, this study did not explore potential interaction effects between variables. Previous research has suggested that significant interactions may exist between factors such as sex and age or occupational environment, which can influence heavy metal exposure levels. A comprehensive review indicates that males and females exhibit different accumulation and toxicological responses to long-term exposure to Pb, Hg, Cd, and As, likely attributable to divergent metabolic pathways and hormonal regulation (34). Consequently, future studies should consider incorporating interaction terms (e.g., age × gender, occupation × region) into regression models to unravel the complexity of exposure pathways.

This study delineates high-risk industries, geographic hotspots, and vulnerable subpopulations for heavy metal exposure among Hunan’s occupational cohorts, informing region- and industry-specific regulatory strategies. Evidence-based measures include: implementing stricter emission standards and OELs for high-exposure industries (e.g., mining, manufacturing); enhancing environmental remediation and occupational hygiene supervision in high-burden regions (e.g., Chang-Zhu-Tan, Xiangnan); and establishing regular health surveillance with preventive mechanisms for high-risk groups. Such targeted approaches would reduce occupational metal exposure, mitigate disease burden, and improve workforce health outcomes.

## 6 Conclusion

Occupational populations in Hunan Province exhibit heterogeneous heavy metal exposure profiles shaped by industrial activities, geographic factors, and individual behaviors. Sustained biomonitoring, enhanced workplace protections, and longitudinal studies are urgently needed to elucidate exposure-disease relationships and safeguard worker health. Future research should expand geographic coverage and incorporate molecular biomarkers to assess subclinical toxicity pathways. These findings provide an empirical foundation for developing region- and industry-specific occupational health policies, strengthening environmental remediation, and optimizing personal protective interventions.

## Data Availability

The original contributions presented in the study are included in the article/supplementary material, further inquiries can be directed to the corresponding author.
